# Severe cerebral air embolism after CT-guided hook-wire localization – complete recovery and delayed lung resection: A case report

**DOI:** 10.1097/MD.0000000000045710

**Published:** 2025-10-31

**Authors:** Zheng Wang, Shuo Liang, Xike Lu, Xin Li, Daqiang Sun

**Affiliations:** a Department of Thoracic Surgery, Tianjin Chest Hospital (Affiliated Hospital of Tianjin University), Tianjin, China; b Department of Radiology, Tianjin Chest Hospital (Affiliated Hospital of Tianjin University), Tianjin, China.

**Keywords:** case report, cerebral infarction, hook-wire localization, systemic air embolism, video-assisted thoracoscopic surgery

## Abstract

**Rationale:**

Systemic air embolism (SAE) is a rare complication of computed tomography–guided hook-wire localization for lung resection, and no prior cases of severe cerebral SAE with complete recovery followed by successful delayed lung resection have been described. This report highlights the management of a severe SAE case and the approach to delayed surgery in the presence of retained wire.

**Patient concerns:**

A 60-year-old man with an 8-mm pure ground-glass nodule in the lingular segment of the left upper lobe developed sudden loss of consciousness and hemoptysis immediately after uncomplicated hook-wire placement.

**Diagnoses:**

Head computed tomography revealed multiple intravascular gas collections in the left frontal lobe, confirming acute cerebral SAE. Additional imaging demonstrated evolving cytotoxic edema involving the bilateral fronto-parieto-occipital lobes and cerebellum.

**Interventions:**

Emergency endotracheal intubation, high-flow normobaric oxygen, sedation, and temperature control (hypothermia) were initiated. Hyperbaric oxygen therapy was not available due to hemodynamic instability and a fresh pulmonary puncture. Intensive neurorehabilitation followed, with close monitoring and gradual imaging over the following 9 days.

**Outcomes:**

The patient experienced marked functional recovery, regaining fine motor skills and independence in daily activities. Fifty-six days post-event, the retained hook-wire was removed during video-assisted thoracoscopic surgery, and successful resection was performed. Histology confirmed minimally invasive adenocarcinoma with negative margins. No perioperative complications occurred, and the patient was discharged on postoperative day 6 without neurological sequelae.

**Lessons:**

This case highlights the importance of early recognition and management of SAE, the role of neurorehabilitation, and the decision-making regarding retained hook-wire. The use of strict positioning post-puncture and the potential for delayed surgery after significant neurological recovery should be considered. While hyperbaric oxygen therapy remains ideal, rapid high-flow oxygen administration and comprehensive care can lead to favorable outcomes, enabling the safe completion of planned thoracic procedures.

## 1. Introduction

Low-dose computed tomography screening reduces lung-cancer-specific mortality by roughly 20 %.^[1]^ Consequently, preoperative localization techniques such as CT-guided hook-wire placement are increasingly utilized to facilitate precise resection of small or sub-solid nodules.^[2]^ Although the overall complication rate is low, systemic air embolism (SAE) – air entering the pulmonary vein and subsequently the left heart, cerebral or coronary circulation – can be catastrophic. Recent studies focusing on ground-glass–nodule localization report an SAE incidence of ~ 0.5 %,^[3]^ suggesting the risk may be underestimated.

Most published reports describe transient, mild neurological symptoms with good outcomes^[4]^; well-documented courses of severe cerebral SAE with serial neuro-imaging and delayed surgery are uncommon. In our high-volume thoracic unit (>3000 operations/year), this was the first severe cerebral SAE with comprehensive radiologic documentation, including near-complete resolution of intracranial air by ~24 hours. Our experience suggests that (i) serial imaging offers a practical reference trajectory; (ii) when the patient is initially unstable, temporarily retaining a barbed wire until safe surgical exposure can be a risk-balanced choice; and (iii) surgical timing should be gated by robust clinical stability plus imaging recovery, rather than early symptom improvement alone.

## 2. Case presentation

**Figure 1.**
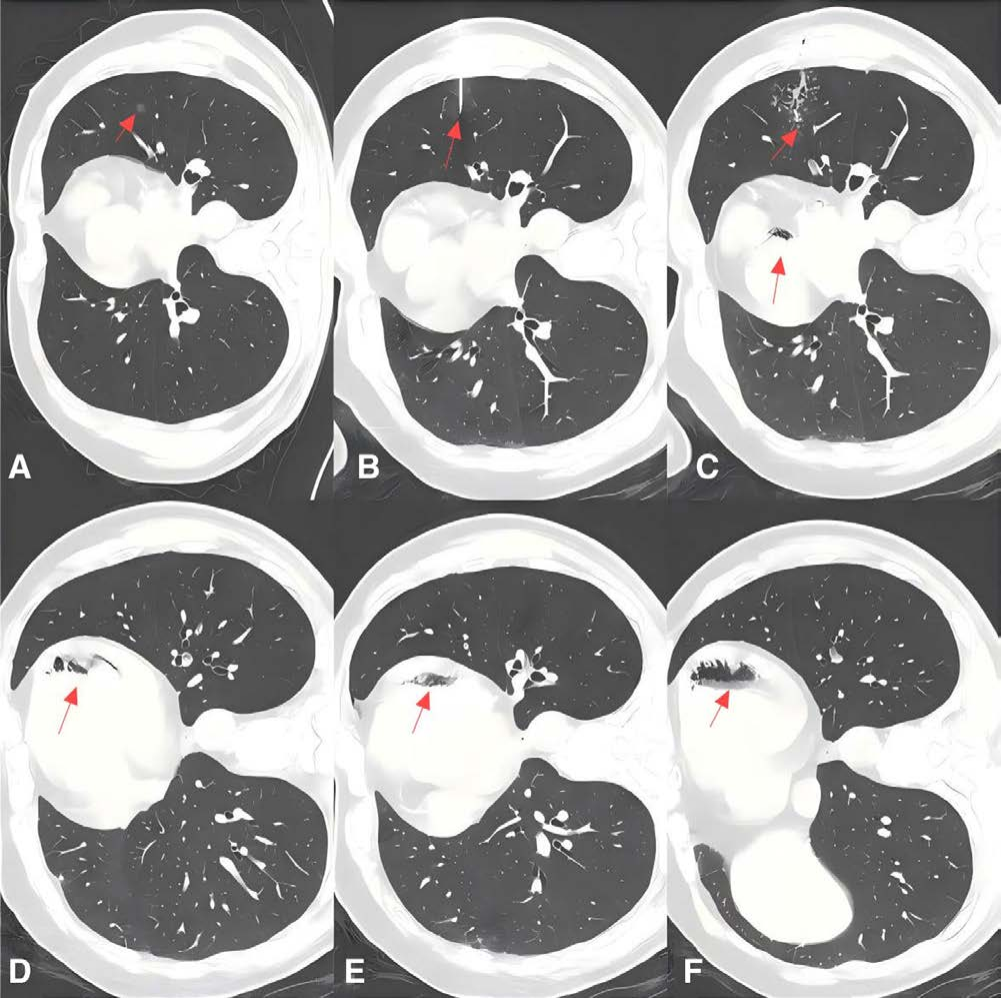
Chest CT on February 25, 2025 (day 0). (A) Baseline scan showing an 8-mm pure ground-glass nodule (white arrow) in the lingular segment of the left upper lobe. (B) Axial slice obtained immediately after hook-wire deployment demonstrates accurate anchoring of the metallic tip (white arrowhead). (C–F) Consecutive slices from the same post-deployment series: scant parenchymal hemorrhage and a small pneumothorax surround the wire track (white arrowheads), while multiple intravascular gas collections are already visible in the aortic root (C), branches of the left superior pulmonary vein (D), the left atrial appendage (E) and the apex and lateral wall of the left ventricle (F) (red arrows). CT = computed tomography.

In February 2025, a 60-year-old man was referred for an incidentally detected 8-mm pure ground-glass nodule in the lingular segment of the left upper lobe (Fig. 1A). Over a year of surveillance the lesion’s size and attenuation had remained unchanged and the patient had stayed asymptomatic; clinical examination, blood tests, spirometry, ECG and echocardiography were all normal. He had no relevant family history of malignancy or neurological disease, and no notable psychosocial or psychological stressors. Because the nodule lay deep in the lingular segment, CT-guided hook-wire localization was planned to secure a tumor-free margin before video-assisted thoracoscopic (VATS) segmentectomy. On February 25,2025, with the patient in the right lateral decubitus position, an experienced thoracic surgeon advanced a 20-G coaxial needle (LN-20-100S™, Pulmon Medical, Nanjing, China) under local anesthesia and deployed the hook-wire precisely (Fig. 1B). The control scan showed accurate anchoring, only scant parenchymal hemorrhage and a small pneumothorax (Fig. 1C).

**Figure 2.**
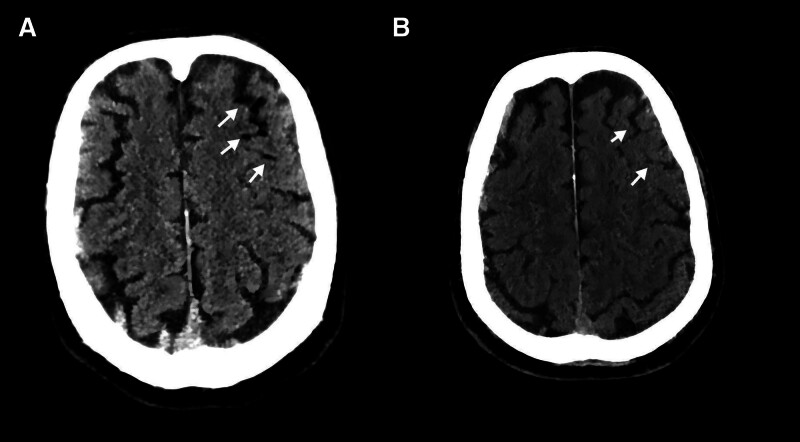
Evolution of intracranial air on non-contrast head CT. (A) Minutes after loss of consciousness, scattered punctate gas densities are present in the sulci of the left-frontal lobe and right parietal lobe (white arrows), confirming cerebral arterial air embolism. (B) Twenty-six hours later, most intracranial gas has been resorbed and cerebral edema has not progressed (white arrows). CT = computed tomography.

While the patient sat up for transfer he suddenly lost consciousness and expectorated blood. Upon the onset of symptoms, pleuropulmonary shock was suspected. However, following the administration of 10 mg dexamethasone, no clinical improvement was observed, prompting the need for emergent intubation and mechanical ventilation. Careful review of the same CT series revealed multiple gas collections in the aortic root, branches of the left superior pulmonary vein, the left atrial appendage and the apex and lateral wall of the left ventricle (Fig. 1C–F, red arrows), indicating that air had already entered the systemic circulation. A head CT obtained minutes later demonstrated scattered punctate gas densities in the left-frontal and right parietal sulci (Fig. 2A, white arrows); cerebral arterial air embolism was diagnosed, and the patient was transferred to the ICU for ventilator-assisted support with metabolic suppression (“hibernation” sedation), targeted temperature management primarily to prevent fever (surface cooling with an ice blanket plus adjunctive ice–saline gastric lavage; hypothermia was intended at 35 °C but not achieved, nadir ≈ 36.5 °C), and comprehensive organ support. After neurology consultation, mannitol was administered q8h for the first 4 days; upon recovery of consciousness, maintenance therapy was switched to glycerol fructose once daily. Head elevation and strict euvolemia were maintained throughout. Repeat CT the following day showed partial resorption of intracranial air without progression of edema (Fig. 2B). Forty-eight hours after onset he regained consciousness: motor strength was normal on the left, diminished on the right. Right-lower-limb power returned within 8 hours, but right-upper-limb recovery lagged, so structured neuro-rehabilitation was initiated. After awakening, sedation was de-escalated to remifentanil-based analgesia, and on the following day the cooling blanket and gastric lavage were discontinued for controlled rewarming. On day 4 the intracranial air had almost disappeared and left-frontal edema had lessened, but a new patchy ischemic focus had appeared in the left parietal lobe; clinically, grip strength of the right little and ring fingers remained weak and the hypothenar region was numb.

**Figure 3.**
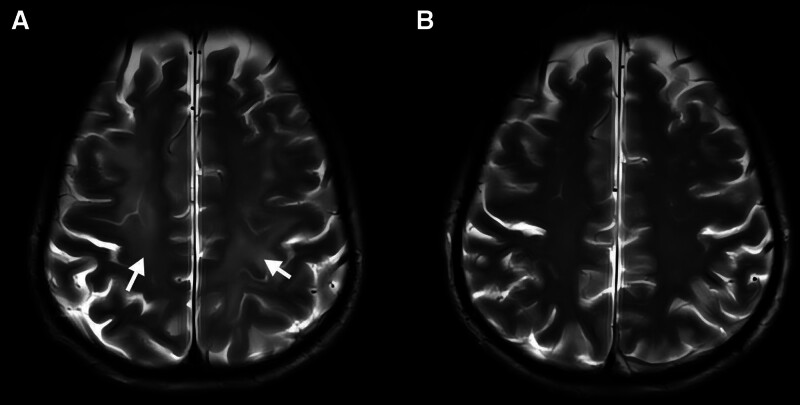
Magnetic-resonance evolution of cerebral injury. (A) Brain MRI on March 5, 2025 (day 8) shows multifocal swelling of the bilateral frontal, parietal and occipital lobes and both cerebellar hemispheres (white arrows). (B) Follow-up MRI on April 8, 2025 (day 43) reveals near-complete resolution of cerebral edema; the previously swollen regions have returned to almost normal signal intensity (white arrows). MRI = magnetic-resonance imaging.

A brain MRI on March 5, 2025 revealed multifocal swelling of both frontal, parietal and occipital lobes as well as the cerebellar hemispheres (Fig. 3A). Home-based rehabilitation continued and the patient received butylphthalide and edaravone for neuroprotection. Follow-up MRI on April 8,2025 showed almost complete resolution of edema (Fig. 3B), and magnetic-resonance angiography demonstrated normal distal cerebral arteries. Fine motor skills in the right hand improved steadily, allowing him to open bottle caps and undo buttons. After multidisciplinary assessment, VATS resection was scheduled for April 22, 2025 using the original hook-wire, which remained securely in place. Intraoperatively multiple firm, calcified lymph nodes surrounded the segmental vessels and bronchus, so a wedge resection with sampling of stations 10 and 11 was performed instead of segmentectomy. Frozen-section and final histology confirmed minimally invasive adenocarcinoma (pT1aN0M0) with clear margins and no nodal metastasis. The postoperative course was uneventful, and the patient was discharged on day 6 without neurological sequelae or other complications. The patient expressed deep gratitude for surviving such a significant complication, humorously attributing his survival to the belief that “the King of Hell did not claim him,” implying that better fortune awaited him. He sincerely acknowledged the proactive and dedicated care provided by the medical team. Initially, the family was frustrated, but over time, they became increasingly supportive, contributing to a successful and positive treatment outcome. The patient adhered to the treatment and rehabilitation plan without experiencing any adverse reactions or intolerance throughout the course of recovery.

## 3. Discussion

SAE is an exceptionally uncommon complication of CT-guided lung-nodule localization. Although only single-case reports have appeared in recent years, its occurrence – however rare – can trigger a rapidly fatal cascade.^[5]^ Once air gains access to the pulmonary veins, it is propelled through the left heart into the cerebral or coronary circulation, where it may provoke cerebral infarction, myocardial infarction and other catastrophic sequelae.

In our patient, the most plausible mechanism was direct entrainment of intrapulmonary air into a pulmonary vein via the needle tract. Pre-procedural CT had shown no cavities, infection, fibrosis or pleural adhesions, yet severe embolism still developed. We surmise that the needle simultaneously traversed a small bronchus and an adjacent vein; when the hook-wire was released and the needle withdrawn, the barbed tip caught both structures and created a persistent bronchovenous fistula. A deep inspiratory effort then lowered venous pressure, drawing a large bolus of air into the vein and onward through the left heart into the systemic arterial tree. Because the patient lay in the right lateral decubitus position, buoyant air preferentially entered the left common carotid artery, explaining the ensuing right-sided deficits. The pathophysiological consequences of SAE encompass ischemia caused by mechanical obstruction, intense vasospasm elicited by intravascular gas, and endothelial injury with secondary platelet activation and microthrombus formation.^[6]^ Symptom severity depends on the destination vessel: tiny bubbles in skeletal muscle or abdominal viscera may be silent, whereas even minute volumes reaching the brain or coronary arteries can prove lethal.

Remarkably, the localization procedure itself was uneventful; the patient collapsed only when he sat upright, implying that a postural change propelled air pooled in the left atrium en masse into the cerebral circulation. Had the air entered slowly or in a dispersed fashion, neurological injury might have been far less dramatic, underscoring the preventive value of meticulous post-procedural positioning. Retrospective scrutiny of the immediate post-puncture CT revealed intracardiac gas that went unrecognized at the time, reinforcing the necessity of whole-lung CT re-scanning and systematic evaluation before any change in posture. Early counter-measures include 100% oxygen, Durant position (left lateral decubitus with slight Trendelenburg when feasible), hemodynamic stabilization, and urgent consideration of hyperbaric oxygen when available. Whole-lung CT re-scanning before posture change is crucial to detect occult intracardiac/pulmonary venous air. In this case only the puncture site received attention; circulatory assessment was overlooked, and an opportunity to avert severe neurological injury was missed.

Hyperbaric oxygen is the treatment of choice for cerebral SAE: by markedly increasing arterial oxygen tension it accelerates bubble dissolution and improves cerebral perfusion, and outcomes are superior when therapy begins within 5 to 7 hours of symptom onset.^[7]^ Our patient, however, was deeply comatose and haemodynamically unstable, with a fresh pulmonary tract that rendered immediate compression therapy a potential trigger for tension pneumothorax; moreover, our center lacks hyperbaric facilities. A multidisciplinary team therefore opted for high-flow normobaric oxygen, sedation, therapeutic hypothermia and comprehensive support. Consciousness returned and neurological function improved markedly, illustrating how swift recognition and timely intervention can yield favorable results even without hyperbaric oxygen.

Prognosis after air-embolism-induced cerebral infarction differs fundamentally from that of thrombotic stroke. The embolic material here is gaseous and can redissolve; high-flow or hyperbaric oxygen gradually eliminates the obstruction. Although acute manifestations may be dramatic, timely treatment often leads to better long-term recovery than is typical for thrombotic infarcts. Because the primary insult is transient mechanical blockage and vasospasm – rather than sustained endothelial damage with full coagulation-cascade activation – neurological restitution is often quicker, as our patient and earlier reports attest.

Finally, the present case shows that even severe SAE does not preclude successful elective resection of the target nodule once rehabilitation is complete. Manufacturers recommend removing a hook-wire within 24 hours, yet here the wire remained in situ for almost 2 months with only minimal tissue reaction. When a major complication arises, systemic stabilization must take precedence; once the patient has recovered, the original localization track can still guide precise, parenchyma-sparing surgery. Our experience therefore supports delayed surgery after full neurological recovery as a safe, feasible and oncologically sound strategy.

## 4. Conclusions

Although SAE is extremely uncommon during CT-guided hook-wire localization, it can precipitate devastating cerebral infarction. Meticulous technique, whole-lung imaging before patient transfer and prudent post-puncture positioning are the cornerstones of prevention. Even in severe cases, prompt high-flow oxygen and comprehensive support usually permit neurological recovery, allowing the planned thoracic operation to proceed safely.

## Author contributions

**Conceptualization:** Zheng Wang.

**Data curation:** Shuo Liang.

**Project administration:** Zheng Wang, Daqiang Sun.

**Resources:** Xike Lu.

**Software:** Shuo Liang.

**Supervision:** Xike Lu, Xin Li.

**Writing – original draft:** Zheng Wang.

**Writing – review & editing:** Xin Li, Daqiang Sun.
